# Apolipoprotein A1: A potential biomarker in the secretome of euploid and aneuploid human embryos

**DOI:** 10.5935/1518-0557.20240106

**Published:** 2025

**Authors:** Mitra Arianmanesh, Fatemeh Hassani, Leila Karimian, Poopak Eftekhari Yazdi, Bahar Movaghar, Bita Ebrahimi, Mostafa Fakhri, Mojtaba Rezazadeh Valojerdi

**Affiliations:** 1 Department of Anatomical Sciences, School of Medicine, Zanjan University of Medical Sciences, Zanjan, Iran; 2 Department of Embryology, Reproductive Biomedicine Research Centre, Royan Institute for Reproductive Biomedicine, ACECR, Tehran, Iran; 3 Department of Genetics, Reproductive Biomedicine Research Centre, Royan Institute for Reproductive Biomedicine, ACECR, Tehran, Iran

**Keywords:** aneuploidy screening, Apolipoprotein A1, blastocyst, conditioned media, non-invasive

## Abstract

**Objective:**

Morphologic assessment of an embryo is a valuable indicator for determining embryo health; however, it does not provide information on the chromosomal status of an embryo. Therefore, this study aimed to investigate the levels of Apolipoprotein A1 secreted by day-5 embryos in the spent media of euploid and aneuploid human embryos.

**Methods:**

This study utilized 131 spent culture media samples from 22 infertile couples who were referred to the fertility clinic of Royan Institute. Following ovulation induction, retrieved oocytes were fertilized by intracytoplasmic sperm injection. For pre-implantation genetic diagnosis, embryos were frozen and thawed on days 2 to 3 and a single blastomere was isolated from each embryo for the assessment of chromosomal abnormalities by fluorescence *in situ* hybridization. Five days after fertilization, the levels of Apolipoprotein A1 were determined in the spent media of normal embryos, aneuploid embryos (with chromosome abnormalities), and the control group (medium without any embryos) using enzyme-linked immunosorbent assay.

**Results:**

The Apolipoprotein A1 levels in the secretome of euploid cleavage-arrested embryos were significantly lower than those in the control group (*p*<0.04). However, Apolipoprotein A1 levels increased significantly in groups of euploid blastocysts, aneuploid cleavage-arrested embryos, aneuploid morulae, and aneuploid blastocysts compared to the control group (*p*<0.04). Furthermore, the Apolipoprotein A1 levels in the spent media of euploid early blastocysts were significantly higher compared to euploid hatching blastocysts and aneuploid blastocysts in the early, mid, and late stages (*p*<0.03).

**Conclusions:**

This study highlights the significant potential of Apolipoprotein A1 as a developmental bi-omarker to distinguish between euploid and aneuploid embryos.

## INTRODUCTION

Despite many improvements in assisted reproductive technology (ART), the success rate remains relatively low (Zargar *et al*., 2021). The selection of a high-quality embryo to transfer to the mother’s uterus is a crucial factor in enhancing the success rate of ART (Zmuidinaite *et al*., 2021). Furthermore, transferring only one embryo helps reduce the risks associated with multiple pregnancies. Selecting a high-quality embryo is a challenging task and needs the expertise of an experienced embryologist or the application of specialized lab techniques (Mains *et al*., 2011). Currently, in most fertility clinics, the assessment of embryo quality is carried out according to the morphological criteria (Ebner *et al*., 2003; Kim *et al*., 2022); however, morphological assessment alone may not identify chromosome aneuploidies (Bielanska *et al*., 2005).

Increasing maternal age is one of the important causes of chromosomal abnormalities in human oocytes and embryos (Sawarkar *et al*., 2021). Approximately 50% of abortions occur during the first trimester of pregnancy due to aneuploidy (Maxwell *et al.,* 2016). Therefore, transferring a euploid embryo (with all 23 pairs of human chromosomes) to the uterus can enhance implantation and live birth rates while reducing the risk of spontaneous pregnancy loss (Sullivan-Pyke & Dokras, 2018).

Pre-implantation genetic diagnosis (PGD) is widely used for aneuploidy screening of an embryo; however, blastomere biopsy for PGD is invasive and may potentially affect the embryo growth and the outcome of in vitro fertilization (IVF) (Chen *et al*., 2021; Navarro-Sánchez *et al*., 2022). In contrast to PGD, omics technologies, including genomics (study of genes), transcriptomics (study of mRNA), proteomics (study of proteins) and metabolomics (study of metabolome) offer a noninvasive approach to aneuploidy screening (Anagnostopoulou *et al*., 2022; Egea *et al*., 2014). Although the number of omics technology is increasing, transcriptomics, proteomics, and metabolomics are widely used in assisted reproductive technologies. Omics techniques can be used to design non-invasive diagnostic methods that are applicable in fertility clinics. For instance, they can be applied for selecting a high quality embryo, particularly in single embryo transfer protocols. Noninvasive analysis of the embryo secretome (proteins and metabolites secreted by an embryo into the surrounding conditioned media) can detect a set of aneuploidy biomarkers, leading to noninvasive viability screening (Cortezzi *et al*., 2011; Schoolcraft *et al*., 2010; Sousa & Monteiro, 2022).

Apolipoprotein A1 (APOA1) is a primary apolipoprotein found in high-density lipoprotein (HDL) and has been identified in human blastocysts (Telford *et al*., 1990). The levels of APOA1 are positively correlated with the quality of human blastocysts and the rate of implantation. Apparently, cholesterol metabolism has a direct relationship with the quality of blastocysts due to the crucial role of cholesterol in the synthesis of cell membranes of the pre-implantation embryo (Mains *et al*., 2011). Given the indication that APOA1 levels are linked to human embryo quality, we hypothesized that the quantity of APOA1 in the secretome may be correlated with aneuploidy. Therefore, this study aimed to investigate the APOA1 protein levels in the secretome of human embryos with chromosome abnormalities (aneuploid) and normal embryos (euploid) in a non-invasive manner.

## MATERIALS AND METHODS

This study was carried out at the Reproductive Biomedicine Research Center at the Royan Institute and involved analyzing conditioned spent media surrounding embryos from PGD candidates who provided informed consent. The research protocol was approved by the Ethics Committee of Royan Institute, Tehran, Iran (reference number: EC/93/1081).

### Patients

**Figure 1:**
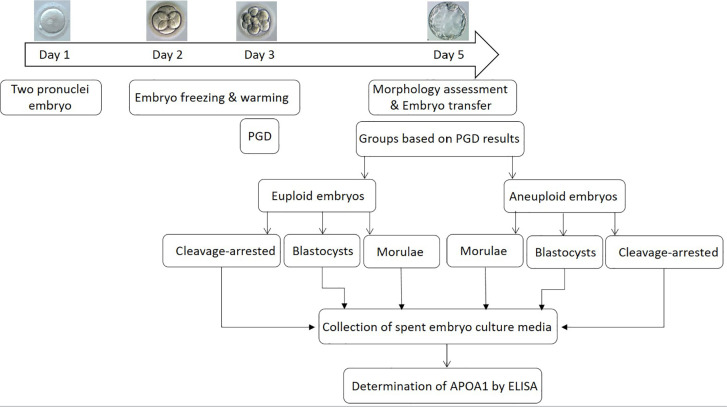
The schematic image indicates the study design in brief.

The study included women aged between 25 and 40 years who were PGD candidates. The exclusion criteria were as follows: (i) endometriosis, (ii) ovarian polycystic syndrome, and (iii) sperms extracted from the testes or epididymis by surgical procedures. The spent media of embryos that were first vitrified and then underwent PGD were included in this study. The experimental design of this study is summarized in a diagram indicated in Figure 1.

### Embryo culture

Human embryos were produced through Intracytoplasmic Sperm Injection (ICSI) after oocyte retrieval using a standard GnRH agonist long protocol as previously described (Rezazadeh Valojerdi *et al*., 2009). The newly formed embryos were cultured in G-1 medium (Vitrolife, Kungsback, Sweden), supplemented with 10% recombinant human serum albumin (rHA, Vitrolife) in an environment of 5% CO_2_ in air at 37°C for 2 - 3 days. On either day 2 or 3, the embryos were cryopreserved and thawed when the endometrium was prepared to receive the embryo (Rezazadeh Valojerdi *et al*., 2009).

For vitrification, embryos were initially incubated in equilibration solution of 7.5% ethylene glycol (EG) (Sigma-Aldrich, Steinleim, Germany) and 7.5% dimethyl sulphoxide (DMSO) (Sigma-Aldrich) in Ham’s F-10 medium supplemented with 20% Albuminal-5 for 5-15 min. Then equilibrated embryos were exposed to the vitrification solution of 15% EG, 15% DMSO and 0.5 M sucrose in Ham’s F-10 medium supplemented with 20% Albuminal-5 for 50-60 s and then loaded on the tip of the cryotop (Kitazato, Japan) and immediately plunged into the liquid nitrogen. For warming, the embryos were incubated in thawing solution (1M sucrose in Ham’s F-10 medium supplemented with 20% Albumin-5) for 50-60 s and then transferred into dilution solution of 0.5 M sucrose for 3min, followed by another dilution solution of 0.25M sucrose for 3min.

Cryopreserved-thawed embryos (72 hours after fertilization) underwent PGD to detect chromosomal abnormalities. For the embryo biopsy procedure, the embryos were incubated in Ca2+/Mg2±free G-PGDTM biopsy medium (Vitrolife, Sweden) for 1-2 minutes. To detect chromosomal abnormalities, fluorescence in situ hybridization (FISH) was carried out on the isolated blastomere (Bazrgar *et al*., 2016). Following the biopsy of one blastomere, the embryos were placed in individual 50 µL droplets of G-2 medium (Vitrolife, Kungsback, Sweden) supplemented with 10% recombinant human serum albumin and incubated until day 5. Simultaneously, a 50 µL droplet of G-2 culture media without any embryo was incubated for 48 hours under the same conditions as a blank control.

### Grading the embryos on day 5 post-fertilization

On day 5 post-fertilization, all embryos were examined under an inverted microscope (Olympus, Japan) by an expert embryologist, and the embryos were classified into three main groups: embryos arrested at the cleavage stage, embryos at the morula stage, and embryos at the blastocyst stage. Blastocysts were also classified into four subgroups: early blastocyst (grade I) where the blastocoel cavity occupied less than 50% of the embryo volume, mid blastocyst (grade II) where the blastocoel cavity filled 50% or more of the embryo volume, late blastocyst (grade III) where the blastocoel cavity filled the embryos and the zona pellucida (ZP) became thinner, and hatching/hatched blastocysts (grade IV) representing embryos that had hatched from the ZP (Hardarson *et al*., 2012).

### Collection of spent embryo culture media

A total of 131 spent embryo culture media samples from 22 patients with an average maternal age of 31.36±1.07 years were collected. After the receipt of PGD results, the normal embryos (euploid) were transferred to the uterus on day 5 post-fertilization, and their spent culture media were collected. Also, the spent embryo culture media of embryos with two or higher chromosomal abnormalities were collected for the study. In total, all spent embryo culture media, where the embryos were incubated for about 48 hours, from both euploid (41 samples) and aneuploid (90 samples) embryos, along with the blank control culture media (media without embryos, 20 samples) were collected and kept individually in labeled cryotubes at -80°C for further analysis.

### Determination of APOA1 levels using the enzyme-linked immunosorbent assay (ELISA) method

The APOA1 levels in spent embryo culture media and blank control media were determined using a quantitative sandwich ELISA kit (ab108803, Abcam Ltd, Cambridge, UK). The minimum detectable dose of APOA1 for this kit was 3 ng/mL. Serial dilutions of the APOA1 protein standard were performed using a diluent to achieve a concentration range from 200 to 3.13 ng in a final volume of 50 µL. The culture media were thawed and diluted to a final volume of 50 µL. Each well in the ELISA plate was coated with polyclonal anti-APOA1 (anti-human APOA1). Also, 50 µL of all standards, diluted samples, and blank control (only diluent) were incubated on the antibody-coated plate at room temperature. All standards, diluted samples, and the blank control were tested in duplicate. After 2 hours, the plate was rinsed, and then the biotinylated APOA1 antibody was added and incubated for 1 hour. Following the plate rinse, the streptavidin conjugate antibody (streptavidin-peroxidase complex) was applied and left to incubate for 30 minutes. Subsequently, the unbound conjugates were washed off. A chromogenic substrate, TMB (3,30,5,50-tetramethylbenzidine), was then used to measure the streptavidin-peroxidase enzymatic reaction. After about 15 minutes, the reaction was halted by adding an acidic stop solution, resulting in a yellow-colored product. The intensity of the yellow color was then measured at 450 nm using a spectrophotometer to determine the APOA1 concentration in all the samples used. Each duplicated media sample was tested three times, resulting in six estimates. These six estimates were analyzed simultaneously to indicate the relative levels of APOA1 compared to the standard curve.

### Statistical analysis

After testing the normality of the data through histograms and statistical tests, such as Shapiro-Wilk and Kolmogorov-Smirnov tests, the normally distributed data were subjected to one-way ANOVA and Bonferroni post-hoc test using SPSS 16 software (IBM, Chicago, USA) to assess the significance of differences. Differences were deemed significant if *p*=0.05, and statistical comparisons between specific groups were carried out using the student’s t-test. The results are expressed as mean±standard error (SE).

## RESULTS

### APOA1 levels in spent culture media of euploid and aneuploid embryos

**Figure 2:**
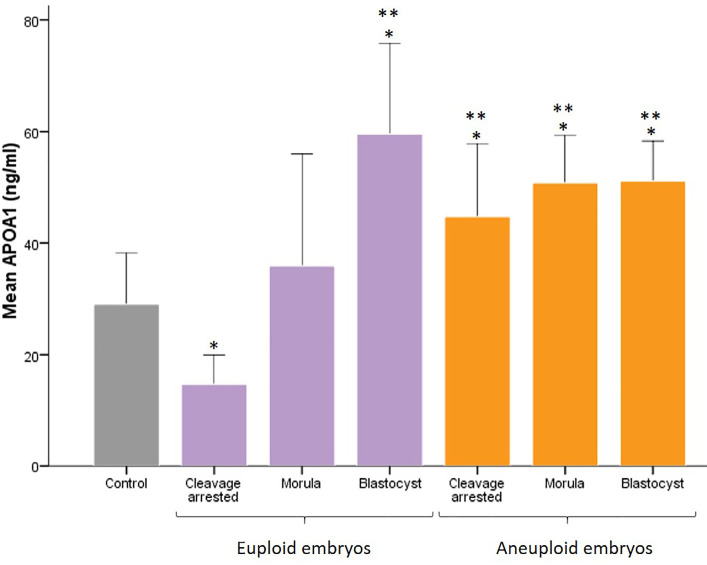
APOA1 level in spent embryo culture media for groups of control (without embryos), euploid embryos and aneuploid embryos. Data are reported as Mean±SE. *: Significant difference between groups (*p*=0.04) *vs*. control. **: Significant difference between groups (*p*=0.02) *vs*. euploid cleavage arrested.

APOA1 levels in all aneuploid embryos, including cleavage-arrested embryos (44.74±6.5ng/mL), morulae (50.74±4.2ng/mL), and blastocysts (51.07±3.6ng/mL), as well as in euploid blastocysts (59.58±8.12ng/mL) were significantly higher than those in the control group (29±4.59ng/mL) (*p*=0.04) (Figure 2). APOA1 levels were significantly lower in euploid cleavage-arrested embryos (14.67±2.63ng/mL) compared to all other groups (*p*=0.02) and the control group (*p*=0.04). However, this reduction was not significant compared to the euploid morulae group (35.86±10.03ng/mL) (Figure 2).

### APOA1 levels in spent culture media of euploid and aneuploid blastocysts at different grades

**Figure 3:**
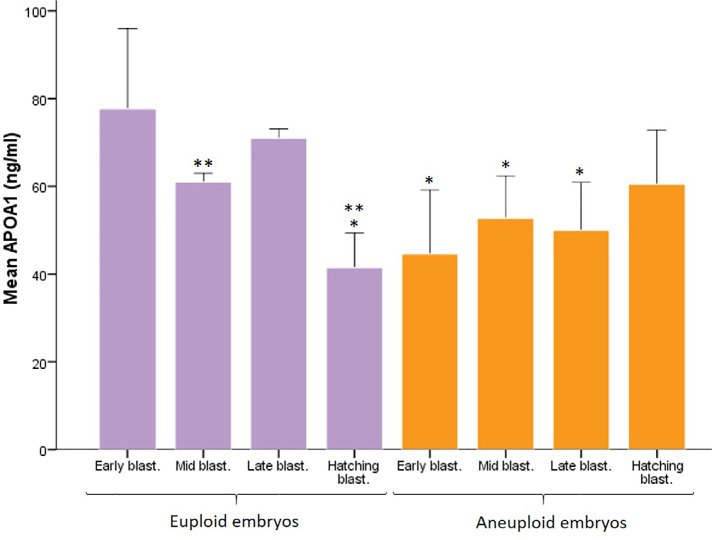
APOA1 level in spent embryo culture media for groups of euploid blastocysts and aneuploid blastocysts at different grades. Data are reported as Mean±SE. *: Significant difference between groups (*p*=0.03) *vs*. euploid early blastocysts. **: Significant difference between groups (*p*=0.03) *vs*. euploid late blastocysts. Blast.: blastocyst.

APOA1 levels were notably higher in euploid early blastocysts (77.67±9.13ng/mL) compared to euploid hatching blastocysts (41.60±3.96ng/mL) and aneuploid blastocysts, including early (44.59±7.2ng/mL), mid (52.75±4.8ng/mL), and late (50±5.43ng/mL) blastocysts (*p=*0.03) (Figure 3). APOA1 levels changed significantly among euploid blastocysts of different grades, with late-stage blastocysts (71±1ng/mL) showing significantly higher levels compared to mid-stage (61±1ng/mL) and hatching blastocysts (41.6±3.96ng/mL) (*p*=0.03) (Figure 3). However, the APOA1 levels did not show significant changes in aneuploid blastocysts across different grades (Figure 3).

## DISCUSSION

Finding a noninvasive, low-cost, and time-consuming method for aneuploidy screening of an embryo can help embryologists select a healthy embryo without any chromosomal abnormalities for embryo transfer. Additionally, a specific biomarker in pre-implantation embryos may guide embryologists to transfer only one high-quality embryo to the endometrium, increasing the chance of embryo implantation and avoiding the risk of multiple gestations. Recently, some molecules including ubiquitin (Katz-Jaffe *et al*., 2006) and lipocalin 1 (McReynolds *et al*., 2011) have been introduced as biomarkers indicating a healthy state of an embryo. However, to date, no distinct biomarker has been found. Indeed, the complexity of embryo development involves many molecules and factors, making it challenging to identify a single potential biomarker for noninvasive aneuploidy screening, therefore a set of biomarkers is required for the selection of a high-quality embryo.

APOA1 is a main apolipoprotein found in high-density lipoprotein (HDL) (Santos *et al*., 2008), which plays an important role in cholesterol metabolism during cell division, cell membrane synthesis, and embryo implantation (Brosens *et al*., 2010). Although there are many studies on APOA1 concerning the female reproductive system, particularly endometrium, there remains insufficient information regarding the synthesis and function of APOA1 in pre-implantation embryos. Given the indication that APOA1 levels are linked to human embryo quality, we hypothesized that the quantity of APOA1 in the secretome may be correlated with aneuploidy.

The reduction in APOA1 in follicular fluid leads to increased embryo fragmentation (Browne *et al*., 2008) and the occurrence of cleavage-arrested embryos (Trigatti *et al*., 1999), indicating the importance of cholesterol and APOA1 in pre-implantation embryo development. In women with recurrent implantation failure (RIF), the level of APOA1 in the endometrium was found to be 4.2 times higher than in healthy women. APOA1 levels decrease in the endometrium during the mid-secretory phase under the influence of LH/hCG and also diminish during embryo implantation (Brosens *et al*., 2010). Therefore, the elevation of APOA1 levels in the secretome of euploid blastocysts, as indicated by the findings of this study, along with the reduction of APOA1 expression in the endometrium during embryo implantation (Brosens *et al*., 2010), may serve as a signaling mechanism. This signaling could potentially aid in the embryo’s interaction with the mother’s endometrium, leading to a more favorable response of the endometrium to the implanting embryo.

Familial APOA1 deficiency causes a remarkable reduction in HDL levels (Bekaert *et al*., 1991; Santos *et al*., 2008), and mice with impaired APOA1 synthesis exhibit very low HDL levels (Williamson *et al*., 1992). Intracellular cholesterol plays a crucial role in cytokinesis during mitosis (Simons & Ikonen, 2000); thus, cholesterol depletion in urchins results in incomplete cytokinesis (Ng *et al*., 2005). Also, cholesterol is vital for embryo development as it aids in energy production through oxidative pathways and supports rapid membrane synthesis during cellular proliferation (Sturmey *et al*., 2006). Therefore, the increase in APOA1 levels in the secretome of euploid blastocysts compared to euploid morulae may be linked to the significance of cholesterol metabolism and phospholipids in pre-implantation embryo development. As the embryo develops from the morula stage (16-32 blastomeres) to the blastocyst stage (100-120 blastomeres), rapid mitosis divisions and a rise in cell numbers occur within only 1-2 days, facilitated by cholesterol metabolism and the synthesis of cell membranes for the newly formed blastomeres. However, minimal and insignificant changes were observed in APOA1 secretion among aneuploid embryos in the current study. Embryos with chromosomal abnormalities likely encounter disruptions in the expression and synthesis of proteins, including APOA1.

Mains *et al*. (2011) reported that the APOA1 levels in the spent embryo culture media of high-quality blastocysts are higher than in low-quality blastocysts, demonstrating that cholesterol metabolism can be an important factor for embryo developmental competence. This observation could also be linked to the antioxidant properties of HDL in the presence of APOA1 (Goulinet & Chapman, 1997; Kontush & Chapman, 2010; Moss *et al*., 2012). Therefore, the increase in APOA1 levels in the secretome of euploid blastocysts compared to aneuploid blastocysts may help in scavenging the free radicals generated during ART procedures (Agarwal & Majzoub, 2017).

The evaluation of human embryo secretome presents challenges because of differences in protocols, reagents, and culture media used in fertility clinics. Many fertility clinics utilize serum protein substitutes to supply the culture media, which contain many undesired proteins, including albumin, potentially leading to discrepancies in secretome assessments (Mains *et al*., 2011). In other words, the accuracy of measurement tools, various embryo culture media, and the actual and precise levels of secreted proteins (varying orders of magnitude) (Ménézo *et al*., 2006; Sargent *et al*., 2007) can result in conflicting data. Virant-Klun *et al*. (2016) reported findings contradicting those of Mains *et al*. (2011). They found that the APOA1 levels in the spent embryo culture media of high-quality blastocysts are lower than low-quality blastocysts, morulae, and degenerated embryos, supporting the findings of Nyalwidhe *et al*. (2013). However, this comparison may not be entirely accurate as they measured APOA1 levels in the secretome of blastocysts, whereas Nyalwidhe *et al*. (2013) measured them in the secretome of day-2 and day-3 embryos.

Nyalwidhe *et al*. (2013) reported a negative correlation between APOA1 levels in the secretome of 2- and 3-day embryos and the quality of embryos and pregnancy rate. This could be attributed to high-quality embryos using more APOA1 from the embryo culture or APOA1 from culture media binding to the zona pellucida (ZP) or/and the embryo surface (Nyalwidhe *et al*. 2013). Human embryo development and protein synthesis are entirely reliant on the mother’s genome until the 4-8 cell stage, after which the human embryo genome becomes active, initiating transcription, translation, and protein synthesis at this point (Jukam *et al*., 2017). APOA1 mRNA has been detected in human blastocysts but not in day-3 human embryos (Telford *et al*., 1990). However, in another study, the APOA1 protein was identified in the conditioned medium of human embryos on the third day of in vitro embryo culture (Foresta *et al*., 2016). The conflicting data may arise due to the application of different techniques in the studies. Before the blastocyst stage, the embryo is not able to synthesize active cholesterol because of the absence of APOA1 protein and the lack of hydroxymethylglutaryl co-enzyme A. Apparently, the oocyte is the only source of cholesterol for the embryo before the blastocyst stage (Fujimoto *et al*., 2010). Therefore, the blastocyst stage could be the best embryo development stage to investigate the levels of APOA1 in embryo secretome. This stage may explain the reasons for the contradictory results between the study by Nyalwidhe *et al*. (2013) and the present study.

The main limitation of the present study is the inability to track the outcome of the embryo implantation and ongoing pregnancies of the embryos whose conditioned medium was used for the study, as multiple embryos were transferred to the uterus. To address this issue, conducting the study with a protocol of single-embryo transfer would be preferable to monitor the outcomes of embryo implantation and pregnancy. Using vitrified/warmed embryos in this research instead of fresh embryos was another limitation of the study, as vitrification/warming can impact embryo viability and may also change the secreted proteins by the embryo. Therefore, using the fresh embryo secretome could help to avoid this problem. A strength of the present study is that the samples were derived from humans and not based on animal research. Furthermore, the study focused on the human embryo secretome in spent culture media rather than on the human embryo itself, thereby adhering to strict ethical standards regarding research on human samples.

In general, finding a noninvasive method for screening aneuploidy in fertility clinics is useful and necessary. However, to achieve a biomarker for aneuploidy, conducting research with a larger sample size and using various types of laboratory techniques, including mass spectrometry, two-dimensional gel electrophoresis, and protein microarray, is suggested. Furthermore, delving deeper into the embryo secretome enhances our understanding of the biological functions of the embryo and the communication between the developing embryo and its surrounding environment. Based on the findings of this study, APOA1 could be a promising candidate to consider as a potential biomarker for aneuploidy screening, although further comprehensive studies are necessary. Given the complexity of embryo development involving numerous molecules and factors, identifying a single potential biomarker for noninvasive aneuploidy screening may prove challenging. It might be more effective to concentrate on a panel of biomarkers rather than relying only on one.
